# CircRNA casein kinase 1 gamma 1 (circ‐CSNK1G1) plays carcinogenic effects in thyroid cancer by acting as miR‐149‐5p sponge and relieving the suppression of miR‐149‐5p on mitogen‐activated protein kinase 1 (MAPK1)

**DOI:** 10.1002/jcla.24188

**Published:** 2022-01-13

**Authors:** Huan Chen, Qin Li, Rong Yi, Baiyun Li, Dongling Xiong, Hui Peng

**Affiliations:** ^1^ Department of Endocrine‐metabolic Yichun People's Hospital Yichun China

**Keywords:** circ‐CSNK1G1, MAPK1, miR‐149‐5p, thyroid cancer

## Abstract

**Background:**

The initiation and development of thyroid cancer may be associated with the deregulation of circular RNAs (circRNAs). The purpose of this work was to explore the role of circRNA casein kinase 1 gamma 1 (circ‐CSNK1G1) in thyroid cancer.

**Methods:**

The expression of circ‐CSNK1G1, miR‐149‐5p, and mitogen‐activated protein kinase 1 (MAPK1) was concluded using quantitative real‐time PCR (qPCR), and the expression of MAPK1 protein was detected by Western blot assay. Cell viability was monitored by CCK‐8 assay. Cell proliferation was determined by colony formation assay and EdU assay. Cell apoptosis and cycle were checked by flow cytometry assay. Cell invasion was determined by transwell assay. The predicted binding relationship between miR‐149‐5p and circ‐CSNK1G1 or MAPK1 was verified by dual‐luciferase reporter assay. The role of circ‐CSNK1G1 in vivo was determined by establishing animal models.

**Results:**

The present work discovered the upregulation of circ‐CSNK1G1 in tumor tissues of thyroid cancer. In function, circ‐CSNK1G1 knockdown inhibited proliferation, survival, and invasion in cancer cells, and tumor growth in mouse models. MiR‐149‐5p was a target of circ‐CSNK1G1, and the anti‐tumor effects of circ‐CSNK1G1 knockdown were abolished by miR‐149‐5p downregulation. In addition, miR‐149‐5p directly targeted MAPK1, and miR‐149‐5p restoration‐inhibited cell proliferation and invasion were recovered by MAPK1 overexpression.

**Conclusion:**

Circ‐CSNK1G1 acted as miR‐149‐5p to relieve the inhibition of miR‐149‐5p on MAPK1, thus promoting the malignant development of thyroid cancer.

## Graphical abstract

Circ‐CSNK1G1 was overexpressed in thyroid cancer. Circ‐CSNK1G1 promoted the malignant development of thyroid cancer partially by activating MAPK1 via competitively targeting miR‐149‐5p

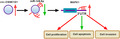

## 1 INTRODUCTION

Thyroid cancer accounts for 3.4% of all newly diagnosed cases of cancer every year in the USA in 2017.^1^ Papillary thyroid cancer is the most common histological subtype of thyroid cancer, accounting for over 80% of new cases.^2^ In general, the relative 5 years survival rate of thyroid cancer has always been the highest among all cancers.^3^ Even so, there are still some patients who do not respond to the usual standard of treatment, and many cases will relapse within 10 years, leading to death.^4^ Thus, a comprehensive understanding of thyroid cancer pathogenesis is crucial.

Along with the boom of circRNA sequencing, the dysregulation of various circRNAs has been exposed in tumor tissues or cells. Accumulating studies have published that circRNAs played wide functions in cancer cell growth, survival, and migration.^5^ In thyroid cancer, the published literature showed that the increased expression of circRNA_102171, circZFR, and circNEK6 in thyroid cancer was related to promoting cell proliferation, invasion, and migration,^6^, ^7^, ^8^ highlighting the vital role of circRNAs in the aggression of thyroid cancer. In view of the high stability, circRNAs are appropriate for clinical diagnosis and treatment as biomarkers in cancers.^9^ However, research on the role of circRNAs in thyroid cancer is just the tip of an iceberg. There are still numerous circRNAs with unclear functions in thyroid cancer, such as circ_0001955. Circ_0001955 is produced from casein kinase 1 gamma 1 (CSNK1G1) by back‐splicing, also terming as circ‐CSNK1G1. It was shown to be highly regulated in papillary thyroid cancer tumor tissues compared with normal tissues through microarray analysis by the public GEO database (accession: GSE93522), hinting that circ‐CSNK1G1 was involved in thyroid cancer development. Nonetheless, the detailed functions of circ‐CSNK1G1 in thyroid cancer were still unclear.

It is widely demonstrated that circRNAs, as competing endogenous RNAs (ceRNAs), compete for microRNA (miRNA) binding sites to relieve the inhibition on downstream target genes.^10^ Public bioinformatics database predicts that circ‐CSNK1G1 harbors binding sites with miR‐149‐5p. MiR‐149‐5p was previously announced to be involved in medullary thyroid cancer as a tumor suppressor.^11^ It was unknown whether circ‐CSNK1G1 functioned by targeting miR‐149‐5p in thyroid cancer. In addition, public bioinformatics database also shows that miR‐149‐5p binds to the 3’ untranslated region (3'UTR) of mitogen‐activated protein kinase 1 (MAPK1), hinting the potential binding between MAPK1 and miR‐149‐5p. MAPK1 was a well‐known oncogene in various cancers, including thyroid cancer.^12^ It was unclear whether miR‐149‐5p played anti‐tumor roles in thyroid cancer by depleting MAPK1.

Our current work was the first to investigate the detailed role of circ‐CSNK1G1 in thyroid cancer. Mechanically, we clarified that circ‐CSNK1G1 served as a ceRNA to compete with MAPK1 for miR‐149‐5p binding site. This work intended to determine the role of circ‐CSNK1G1 in thyroid cancer and provide a mechanism to explain its function.

## 2 MATERIALS AND METHODS

### 2.1 Clinical samples

Patients with thyroid cancer were newly diagnosed and surgically treated at the Yichun People's Hospital. Clinical tissues were frozen after excision and stored at −80°C conditions. Patients who had received any therapies (chemotherapy, radiotherapy, or others) prior to surgery and patients diagnosed with other types of cancer and severe systemic infectious diseases were excluded from this study. A total of 47 pairs of tumor tissues and matched normal tissues were used in this study and respectively confirmed by pathological diagnosis. The clinicopathological parameters of patients with thyroid cancer enrolled in this study were displayed in Table 1, such as age, gender, and tumor stage. The use and study of these samples were permitted by patients with written informed consent. This study was approved by the Ethics Committee of Yichun People's Hospital.

**TABLE 1:** Clinicopathological parameters of patients with thyroid cancer (*n* = 47)

Clinicopathological parameters		*n* = 47
Ages	>45	29
	≤45	18
Gender	Male	21
	Female	26
Tumor stage	Ⅰ‐Ⅱ	27
	Ⅲ‐Ⅳ	20
Lymph node metastasis	Negative	25
	Positive	22
Tumor size	≤2 cm	19
	>2 cm	28

### 2.2 Cells and cell culture

Thyroid cancer cell lines, including IHH‐4, HTH83, KTC‐1, SW579, and TPC‐1, were purchased from Bena. SNU‐790, a cell line of thyroid cancer, was purchased from YaJi biological. IHH‐4 cells were kept in DMEM (GIBCO,) with 10% FBS (GIBCO). SW549 cells were cultured in Leibovitz‐15 medium (GIBCO) containing 10% FBS. SNU‐790, HTH83, KTC‐1, and TPC‐1 cells were cultured in RPMI‐1640 medium (GIBCO) with 10% FBS. Non‐cancer cell line, Nthy‐ori 3–1 (Bena), was used as a control and cultured in F‐12K medium (GIBCO) with 10% FBS. Cells were maintained in a 37°C incubator supplemented with 5% CO_2_.

### 2.3 Quantitative real‐time PCR (qPCR)

TRIzol reagent (Invitrogen,) was applied for RNA isolation. Then, total RNA was used for cDNA assemble using the TaqMan MicroRNA Reverse Transcription kit (Applied Biosystems,) or PrimeScript Reverse Transcription Reagent (Takara,) according to the instructions. Afterward, cDNA was used for amplification using the SYBR Green Master PCR mix (Applied Biosystems). The conditions used for qPCR were 95°C for 10 min (1 cycle), 95°C for 15 s, and 60°C for 1 min (35 cycles). Relative expression was normalized by GAPDH or U6 as appropriate, using the 2^−ΔΔCt^ method. Primers used here were shown below:

circ‐CSNK1G1, F: 5'‐GGACCCTCTTCACAGACCTC‐3' and R: 5'‐GGAGACCTTCACCTGATTTCG‐3'; CSNK1G1, F: 5'‐TGGGTAGAGAAGCACTTGGC‐3' and R: 5'‐TGTAACCTGGTCCAGCAGTG‐3'; miR‐149‐5p, F: 5'‐CGTCTGGCTCCGTGTCTTC‐3' and R: 5'‐AGTGCAGGGTCCGAGGTATT‐3'; MAPK1, F: 5'‐TCCTTTGAGCCGTTTGGAGG‐3' and R: 5'‐TACATACTGCCGCAGGTCAC‐3'; U6, F: 5'‐CTCGCTTCGGCAGCACA‐3' and R: 5'‐AACGCTTCACGAATTTGCGT‐3'; GAPDH, F: 5'‐GGAGTCCACTGGCGTCTTCA‐3' and R: 5'‐GGTTCACACCCATGACGAAC‐3'.

### 2.4 RNase R treatment

SW579 and TPC‐1 cells‐derived total RNA was treated with RNase R (2 U/μg; Epicentre,) for 20 min at 37°C conditions. Total RNA was then examined as abovementioned.

### 2.5 Cell transfection

Small interference RNA (siRNA) for circ‐CSNK1G1 (si‐circ‐CSNK1G1) and its scramble negative control (si‐NC), circ‐CSNK1G1 overexpression fusion vector (circ‐CSNK1G1), and its blank vector control (PCD5‐ciR) were all constructed by Geneseed. Mimics for miR‐149‐5p (miR‐149‐5p) and its negative control (miR‐NC), inhibitor for miR‐149‐5p (anti‐miR‐149‐5p) and its negative control (anti‐miR‐NC) were all purchased from Ribobio. MAPK1 overexpression construct (MAPK1) and blank vector control (pcDNA) were assembled by Sangon Biotech. Oligonucleotides or vectors were used for cell transfection using Lipofectamine 3000 reagent (Invitrogen).

### 2.6 CCK‐8 assay

Cells in complete medium were put into 96‐well plates (5 × l0^3^ cells/well) and cultured for 24 h. After incubation, 10 μl CCK‐8 reagent (Sigma,) was used to treat cells for 2 h. Absorbance (450 nm) was measured for each sample using a microplate reader (BioTek, Biotek Winooski,).

### 2.7 Colony formation assay

Cells in complete medium were plated into 6‐well plates (200 cells/well) and continuingly cultured in a 37°C incubator supplemented with 5% CO_2_ for 2 weeks. Then, cell colonies were fixed using methanol and stained with 0.1% crystal violet. The images of cell colonies were captured under a light microscope (Leica,).

### 2.8 EdU assay

Utilizing Cell‐Light EdU Apollo567 Kit (Ribobio) according to the protocol, the number of EdU‐positive cells was identified. In brief, cells were cultured EdU medium for 4 h, fixed by 4% paraformaldehyde, and next stained by Apollo567 and DAPI. Images were taken with the use of a fluorescence microscope (Leica).

### 2.9 Flow cytometry assay

Cells were cultured for 48 h and then collected. After re‐suspending in binding buffer (1 × 10^6^ cells/ml), cells were next stained with Annexin V‐FITC (Beyotime) and propidium iodide (PI; Beyotime). Cell apoptosis was ensured by a FACS Calibur flow cytometer (BD Biosciences,).

Also by flow cytometry analysis, cell cycle was checked using a Cell Cycle Analysis Kit (Beyotime). Cells were suspended with PBS and then fixed with 70% cooled ethanol. Next, cells were exposed to PI staining buffer containing RNase A, followed by detection under a flow cytometer.

### 2.10 Transwell assay

To assess cell invasion, cells (5 × 10^4^) suspended in serum‐depleted culture medium were transported to the upper chamber of transwell chambers (24‐well; Corning Incorporated, Corning,) pre‐coated with Matrigel (BD Biosciences). Cell culture medium containing 10% FBS, served as a chemoattractant, was supplemented into the lower chambers. After 24 h‐incubation at 37°C, cells invaded to the low surface were fixed using methanol and stained with 0.1% crystal violet. The images of cell morphology and the number of invaded cells in random 5 fields were recorded using an inverted microscope (×100; Leica).

### 2.11 Western blot

Total protein was extracted using RIPA reagent (Beyotime). Western blot was implemented in accordance with the method in a previous study.^13^ The antibodies, including anti‐CyclinD1 (dilution: 1/100; ab16663; Abcam,), anti‐MMP9 (dilution: 1/2000; ab76003; Abcam), anti‐MAPK1 (dilution: 1/1000; AP7501a; Abcepta Biotech), anti‐GAPDH (dilution: 1/2500; ab9485; Abcam), and Goat Anti‐Rabbit IgG H&L (HRP) (dilution: 1/20000; ab205718; Abcam), were used in this study.

### 2.12 Dual‐luciferase reporter assay

According to the predicted wild‐type (WT) binding sites between circ‐CSNK1G1 and miR‐149‐5p by Circinteractome (https://circinteractome.nia.nih.gov/), the mutant (MUT) sequence fragment of circ‐CSNK1G1 was designed. According to the predicted WT binding sites between miR‐149‐5p and MAPK1 3'UTR by Starbase (http://starbase.sysu.edu.cn/), the MUT sequence fragment of MAPK1 3'UTR was designed. Subsequently, luciferase reporter plasmids, including WT‐circ‐CSNK1G1, MUT‐circ‐CSNK1G1, WT‐MAPK1 3'UTR, and MUT‐MAPK1 3'UTR, were constructed by Sangon Biotech. These reporter plasmids singly transfected with miR‐149‐5p mimic or miR‐NC into SW579 and TPC‐1 cells. After incubation for 48 h, luciferase activity was examined with the application of dual‐luciferase reporter assay system (Promega,).

### 2.13 Animal models

The procedures of animal study were approved by Yichun People's Hospital. A total of 12 nude mice (Balb/c, 6‐week‐old) were bought from Vital River Laboratory Animal and raised in standard conditions. Lentivirus suspensions of short hairpin RNA targeting circ‐CSNK1G1 (sh‐circ‐CSNK1G1) or sh‐NC were provided by Geneseed. TPC‐1 cells were infected with sh‐circ‐CSNK1G1 lentivirus suspensions for stable circ‐CSNK1G1 knockdown. Then, nude mice were subcutaneously injected with the infected cells (2 × 10^6^ cells per mouse) to induce tumor growth (*n* = 6 per group). During tumor growth, tumor volume (length × width^2^ × 0.5) was measured every three days. Tumors were allowed to grow 22 days, and all mice were killed for tumor excision.

### 2.14 Immunohistochemical (IHC) analysis

After formalin fixation and paraffin embedding, tissue sections (3 μm thick) were prepared. Tissue sections were deparaffinized and dehydrated, followed by section repairs by microwave antigen using citric acid buffer solution. Next, sections were incubated with the primary antibodies (4°C, overnight), including anti‐CyclinD1, anti‐MMP9, and anti‐MAPK1. After that, sections were probed with Goat Anti‐Rabbit IgG H&L (HRP) at room temperature for 2 h. The sections were stained with 3, 3’‐diaminobenzidine (DAB; Sigma‐Aldrich) and counterstained with hematoxylin. Images were taken under a light microscope (Leica).

### 2.15 Statistical analysis

All analyses were accomplished by GraphPad Prism 7.0 (GraphPad Software,). Data were presented as the mean ± standard deviation. Student's *t* test or analysis of variance was used to compare differences in different groups. Pearson correlation analysis was performed to analyze the linear correlation between two sets. All experiments contained 3 repetitions for each group. *p* < 0.05 was believed as statistical significance.

## 3 RESULTS

### 3.1 Circ‐CSNK1G1 was overexpressed in tumor tissues and cell lines of thyroid cancer

**FIGURE 1:**
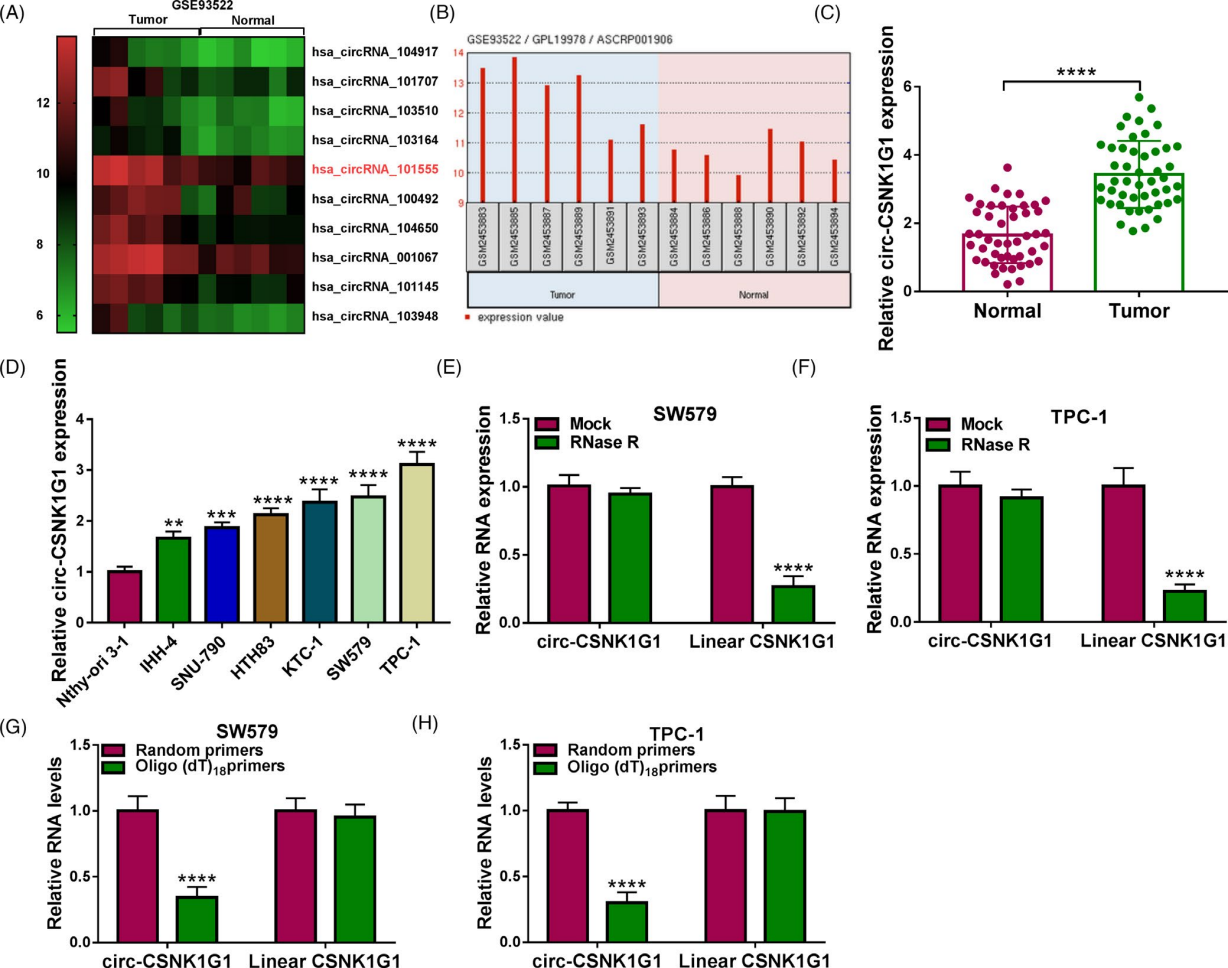
Expression of circ‐CSNK1G1 was increased in tumor tissues and cells of thyroid cancer. (A) The data of differently expressed circRNAs were obtained from the public database (GEO: GSE93522). (B) The expression of circ‐CSNK1G1 in tumor tissues and paired normal tissues from GSE93522. (C) The expression of circ‐CSNK1G1 in our clinical tumor tissues and normal tissues was detected by qPCR. (D) The expression of circ‐CSNK1G1 in cancer cell lines and non‐cancer cells was detected by qPCR. (E and F) The stability of circ‐CSNK1G1 and linear CSNK1G1 was detected by RNase R. (G and H) The circularity of circ‐CSNK1G1 was identified using oligo(dT)_18_ primers. **p < 0.01, ***p < 0.001 and ****p < 0.0001

The data from GSE3522 dataset (a circRNA expression profile) showed that has_circRNA_101555 (circ‐CSNK1G1) was one of the forcefully expressed circRNAs in thyroid cancer tissues compared with normal tissues (Figure 1A and Figure 1B). Then, we found that circ‐CSNK1G1 expression was strikingly higher in tumor tissues of thyroid cancer than that in matched normal tissues (Figure 1C). Besides, circ‐CSNK1G1 expression was also significantly heightened in IHH‐4, NIM, SW579, and TPC‐1 cells relative to Nthy‐ori 3–1 cells (Figure 1D). Moreover, we found that circ‐CSNK1G1 was stable compared with its linear transcript because it was resistant to RNase R digestion (Figure 1E and 1F). Compared with linear CSNK1G1, circ‐CSNK1G1 could not be amplified by oligo(dT)_18_ primers, suggesting that circCSNK1G1 had no 3’ and 5’ tails in structure (Figure 1G and 1H). Figure 1 mainly showed that circ‐CSNK1G1 was abnormally upregulated in thyroid cancer tissues and cells.

### 3.2 Circ‐CSNK1G1 downregulation restrained SW579 and TPC‐1 cell proliferation, survival, and invasion

**FIGURE 2:**
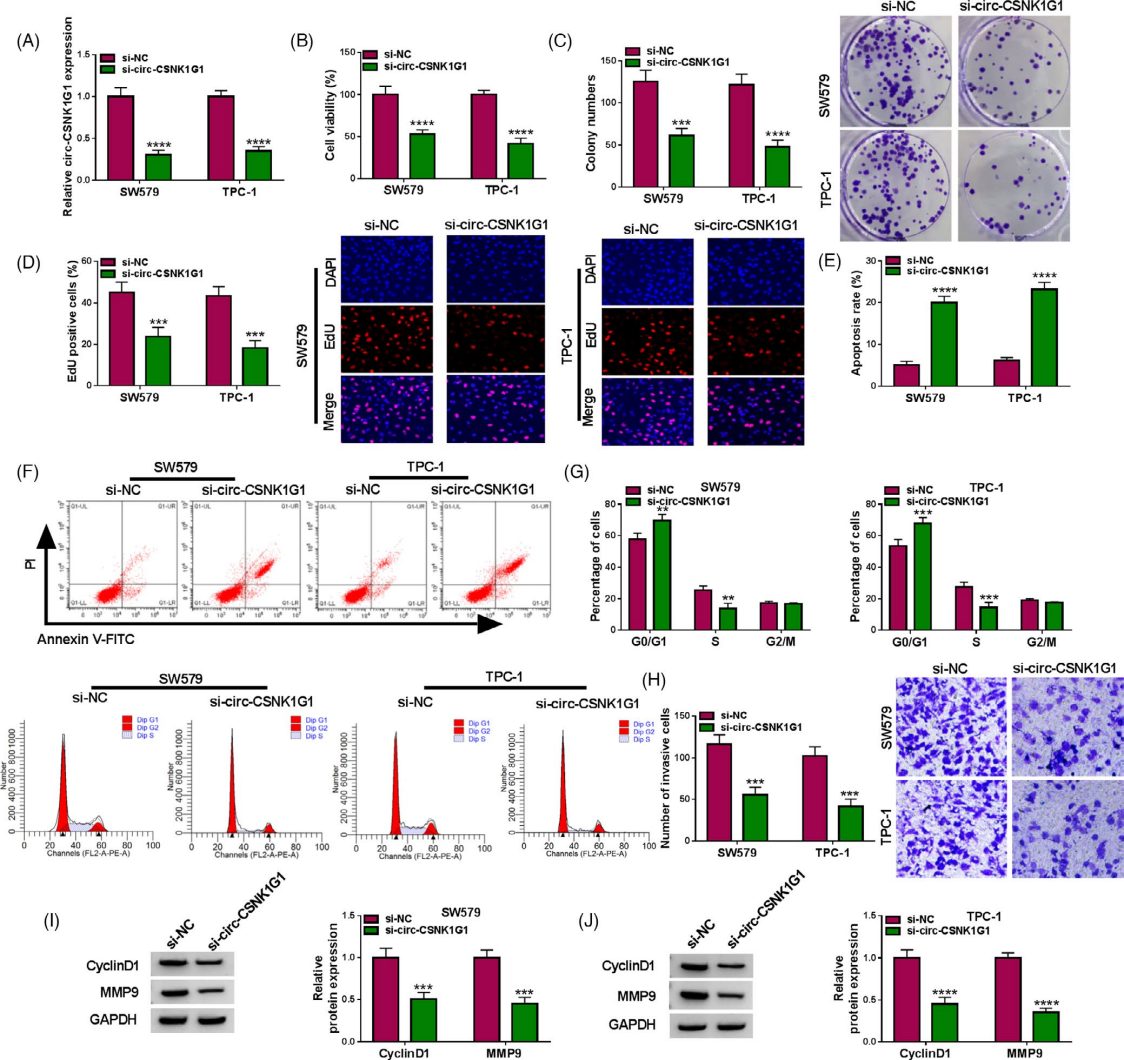
Circ‐CSNK1G1 knockdown‐suppressed thyroid cancer cell proliferation, survival, and invasion. (A) The expression of circ‐CSNK1G1 in SW579 and TPC‐1 cells after circ‐CSNK1G1 knockdown was detected by qPCR. Then, in SW579 and TPC‐1 cells with circ‐CSNK1G1 knockdown, (B) cell viability was detected by CCK‐8 assay. (C) Cell colony formation ability was determined by colony formation assay. (D) Cell proliferation was assessed by EdU assay. (E and F) Cell apoptosis was monitored by flow cytometry assay. (G) Cell cycle was checked by flow cytometry assay. (H) Cell invasion was checked by transwell assay. (I and J) The protein levels of CyclinD1 and MMP9 were measured by Western blot. ***p < 0.001 and ****p < 0.0001

Considering that the expression of circ‐CSNK1G1 was reinforced in SW579 and TPC‐1 cells, we reduced circ‐CSNK1G1 expression in SW579 and TPC‐1 cells by transfecting si‐circ‐CSNK1G1 (Figure 2A). The data from CCK‐8 assay presented that cell viability was notably decreased in SW579 and TPC‐1 cells after circ‐CSNK1G1 knockdown (Figure 2B). The data showed that circ‐CSNK1G1 knockdown impaired the number of colonies and reduced the number of EdU‐positive cells (Figure 2C and 2D). Flow cytometry assay found that circ‐CSNK1G1 knockdown enhanced the number of apoptotic cells (Figure 2E and 2F) and arrested cell cycle at the G0/G1 stage (Figure 2G). Transwell assay was conducted to assess cell invasion, and the capacity of cell invasion was significantly decreased in SW579 and TPC‐1 cells after circ‐CSNK1G1 knockdown (Figure 2H). CyclinD1 is one of the key cell cycle regulators, and MMP9 expression is closely associated with the invasion and migration of tumor cells. Here, we discovered that CyclinD1 and MMP9 were strikingly downregulated in SW579 and TPC‐1 cells transfected with si‐circ‐CSNK1G1 (Figure 2I and 2J). All data in Figure 2 indicated that circ‐CSNK1G1 knockdown inhibited thyroid cancer cell malignant development.

### 3.3 Circ‐CSNK1G1 inhibited the expression of miR‐149‐5p

**FIGURE 3:**
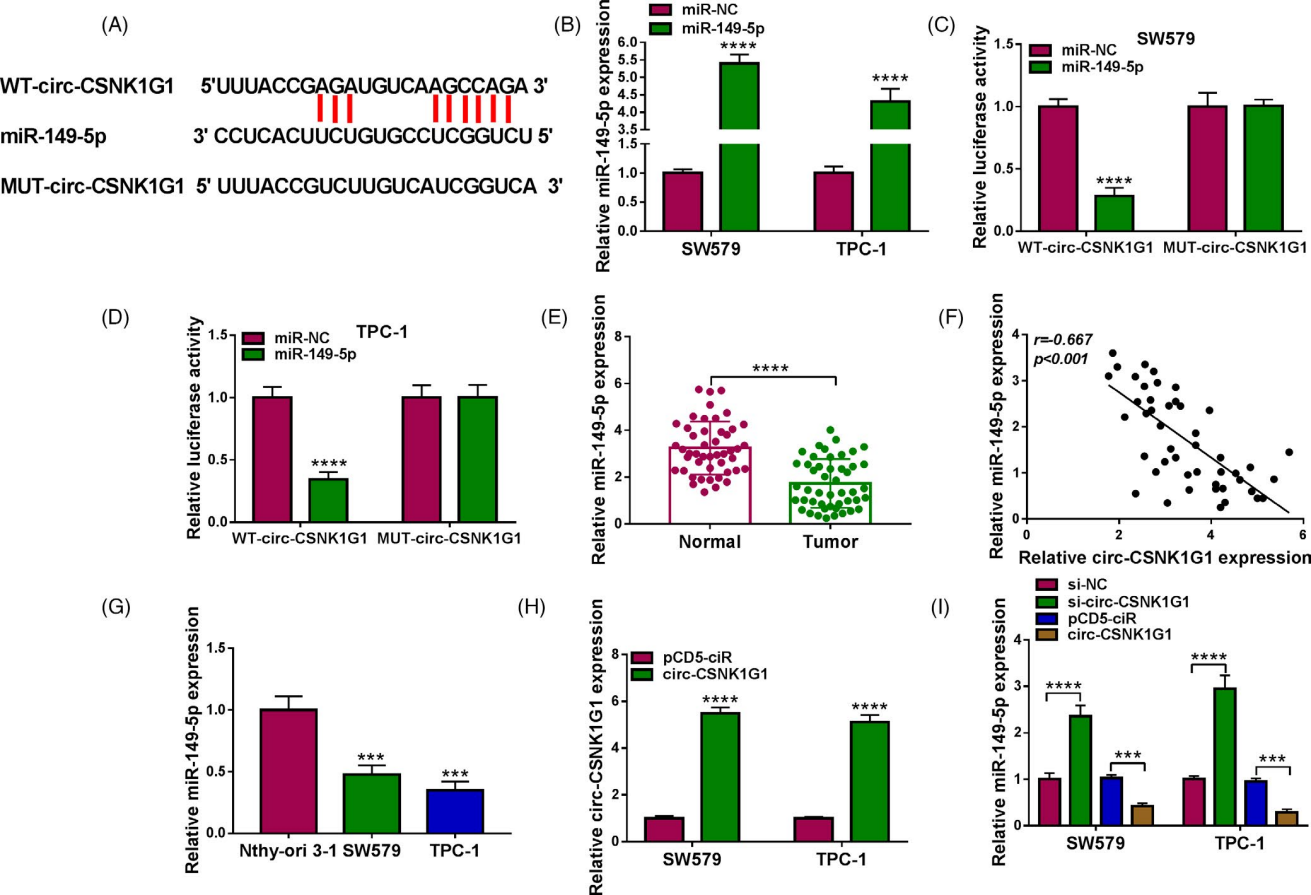
MiR‐149‐5p was a target of circ‐CSNK1G1. (A) The binding site between circ‐CSNK1G1 and miR‐149‐5p was obtained from the public database. (B) The efficiency of miR‐149‐5p mimic was detected by qPCR. (C and D) Their relationship between miR‐149‐5p and circ‐CSNK1G1 was further verified by dual‐luciferase reporter assay. (E) The expression of miR‐149‐5p in tumor tissues and normal tissues was detected by qPCR. (F) The linear relationship between miR‐149‐5p expression and circ‐CSNK1G1 expression was analyzed by Pearson correlation analysis. (G) The expression of miR‐149‐5p in cell lines was detected by qPCR. (H) The efficiency of circ‐CSNK1G1 overexpression was checked by qPCR. (I) The expression of miR‐149‐5p in SW579 and TPC‐1 cells with circ‐CSNK1G1 knockdown or overexpression was detected by qPCR. ***p < 0.001 and ****p < 0.0001

Bioinformatics tool presented that circ‐CSNK1G1 interacted with miR‐149‐5p via several binding sites, hinting that miR‐149‐5p was putatively targeted by circ‐CSNK1G1 (Figure 3A). We next verify the binding between circ‐CSNK1G1 and miR‐149‐5p. MiR‐149‐5p expression was notably enhanced in SW579 and TPC‐1 cells with the transfection of miR‐149‐5p (Figure 3B). Luciferase activity was significantly decreased in SW579 and TPC‐1 cells with miR‐149‐5p and WT‐circ‐CSNK1G1 cotransfection (Figure 3C and 3D), verifying the binding between circ‐CSNK1G1 and miR‐149‐5p. The expression of miR‐149‐5p was notably declined in tumor tissues of thyroid cancer (Figure 3E). Besides, miR‐149‐5p expression was negatively correlated with circ‐CSNK1G1 expression in tumor tissues (Figure 3F). Next, we observed that miR‐149‐5p level was also notably lower in SW579 and TPC‐1 cells than that in Nthy‐ori 3–1 cells (Figure 3G). For circ‐CSNK1G1 overexpression, circ‐CSNK1G1 level was notably increased in SW579 and TPC‐1 cells transfected with circ‐CSNK1G1 compared with pCD5‐ciR (Figure 3H). Interestingly, the expression of miR‐149‐5p was markedly enhanced in SW579 and TPC‐1 cells with circ‐CSNK1G1 knockdown but decreased in cells with circ‐CSNK1G1 overexpression (Figure 3I). These views ensured that miR‐149‐5p was a target of circ‐CSNK1G1.

### 3.4 Circ‐CSNK1G1 knockdown inhibited thyroid cancer cell malignant development by enriching miR‐149‐5p

**FIGURE 4:**
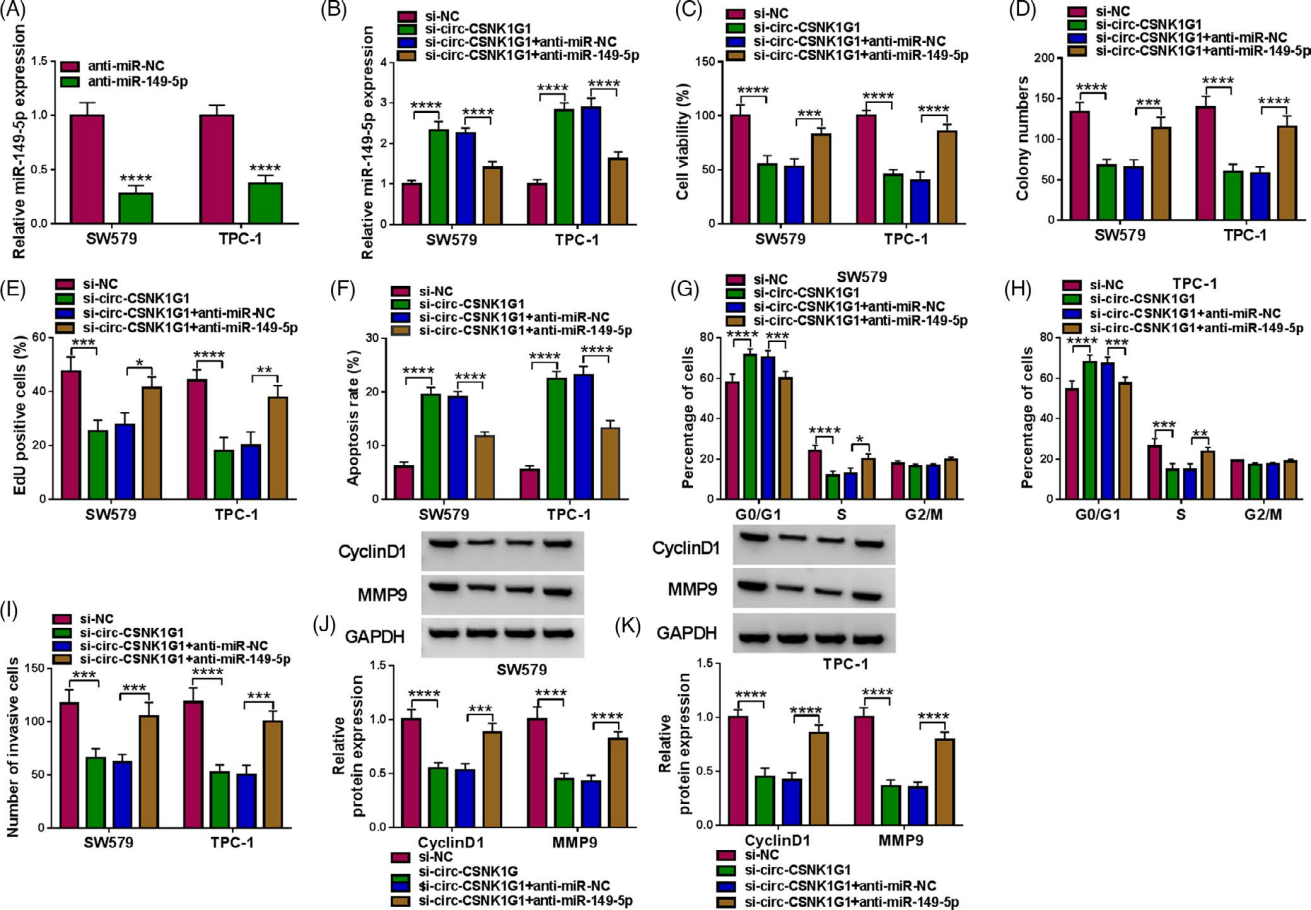
Circ‐CSNK1G1 knockdown‐suppressed thyroid cancer cell proliferation, survival, and invasion were restored by miR‐149‐5p inhibition. (A) The efficiency of miR‐149‐5p inhibitor was checked by qPCR. (B) The expression of miR‐149‐5p in SW579 and TPC‐1 cells transfected with si‐circ‐CSNK1G1 or si‐circ‐CSNK1G1+anti‐miR‐149‐5p was measured by qPCR. In these transfected cells, (C) cell viability was checked by CCK‐8 assay. (D) The ability of colony formation was checked by colony formation assay. (E) Cell proliferation was assessed by EdU assay. (F) Cell apoptosis was detected by flow cytometry assay. (G and H) Cell cycle was investigated by flow cytometry assay. (I) Cell invasion was checked by transwell assay. (J and K) The protein levels of CyclinD1 and MMP9 were detected by Western blot. *p < 0.05, **p < 0.01, ***p < 0.001 and ****p < 0.0001

To validate whether circ‐CSNK1G1 interacted with miR‐149‐5p to play effects in thyroid cancer, rescue experiments were performed. MiR‐149‐5p expression was pronouncedly declined in SW579 and TPC‐1 cells transfected with anti‐miR‐149‐5p (Figure 4A). For rescue experiments, miR‐149‐5p expression was notably enhanced in SW579 and TPC‐1 cells harboring si‐circ‐CSNK1G1 but partially weakened in cells harboring si‐circ‐CSNK1G1+anti‐miR‐149‐5p (Figure 4B). In function, circ‐CSNK1G1 knockdown‐blocked cell viability was partially recovered by miR‐149‐5p inhibition (Figure 4C). Through colony formation assay and EdU assay, we observed that the ability of cell proliferation was suppressed by circ‐CSNK1G1 knockdown but partially restored by miR‐149‐5p depletion (Figure 4D and 4E). In addition, circ‐CSNK1G1 knockdown‐induced cancer cell apoptosis and cell cycle arrest were largely relieved by miR‐149‐5p inhibition (Figure 4F–4H). Circ‐CSNK1G1 knockdown‐suppressed cell invasion was promoted by the inhibition of miR‐149‐5p (Figure 4I). The protein levels of CyclinD1 and TPC‐1 were notably declined in SW579 and TPC‐1 cells harboring si‐circ‐CSNK1G1 but largely recovered in SW549 and TPC‐1 cells harboring si‐circ‐CSNK1G1+anti‐miR‐149‐5p (Figure 4J and 4K). These findings indicated that circ‐CSNK1G1 knockdown inhibited thyroid cancer cell malignant development by enriching miR‐149‐5p.

### 3.5 MiR‐149‐5p inhibited MAPK1 expression by binding MAPK1 3'UTR

**FIGURE 5:**
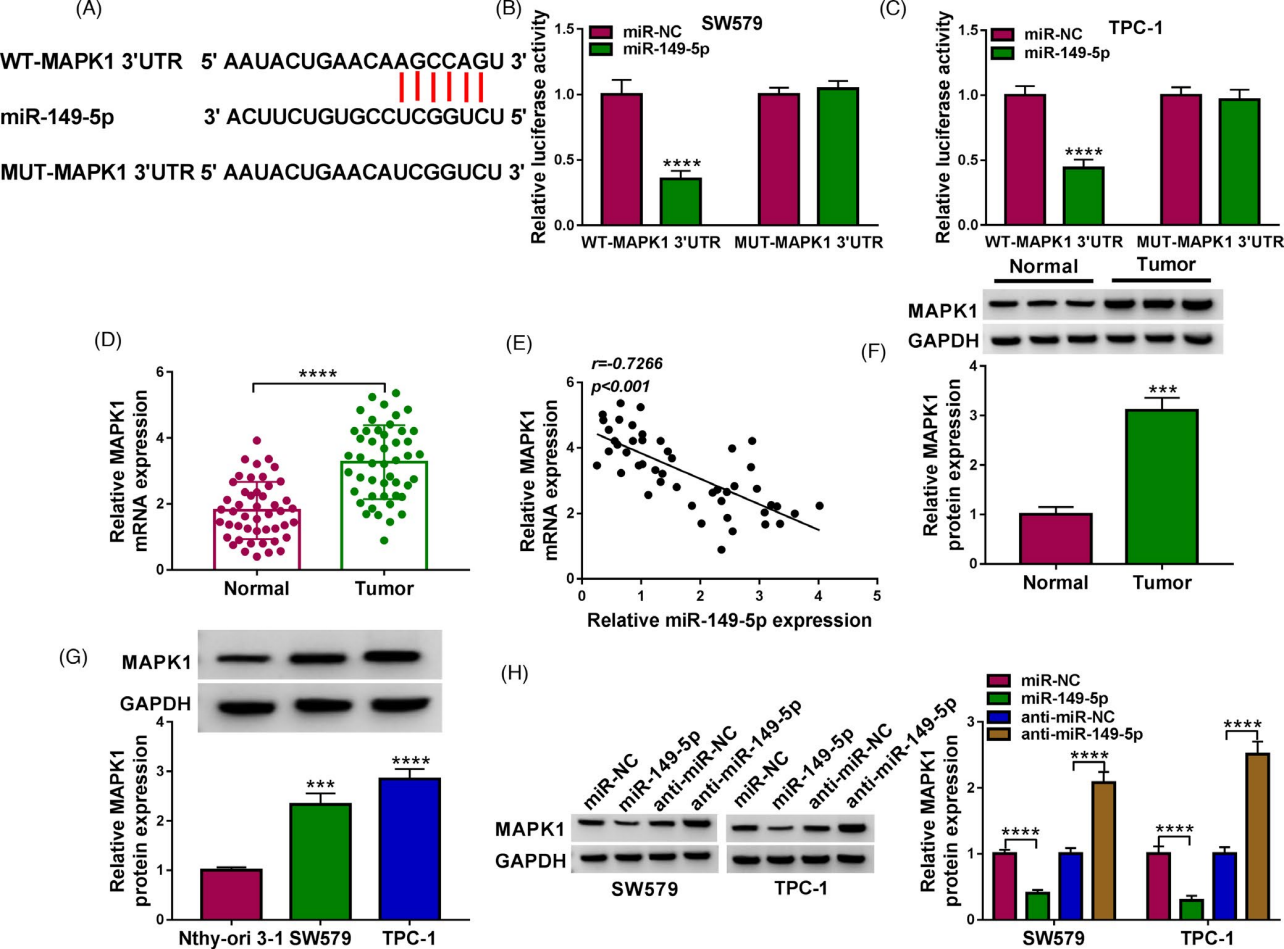
MAPK1 was a target of miR‐149‐5p. (A) The binding site between miR‐149‐5p and MAPK1 3'UTR was obtained from the public database. (B and C) The binding between miR‐149‐5p and MAPK1 was confirmed by dual‐luciferase reporter assay. (D) The expression of MAPK1 mRNA in tumor tissues and normal tissues was detected by qPCR. (E) The linear relationship between miR‐149‐5p expression and MAPK1 expression was analyzed by Pearson correlation analysis. (F) The expression of MAPK1 protein in tumor tissues and normal tissues was detected by Western blot. (G) The expression of MAPK1 protein in cell lines was detected by Western blot. (H) The expression of MAPK1 protein in SW579 and TPC‐1 cells with miR‐149‐5p overexpression or downregulation was detected by Western blot. ***p < 0.001 and ****p < 0.0001

Bioinformatics analysis displayed that miR‐149‐5p interacted with MAPK1 3'UTR via a special binding site (Figure 5A). Luciferase activity was remarkably declined in SW579 and TPC‐1 cells transfected with miR‐149‐5p and WT‐MAPK1 3'UTR (Figure 5B and 5C). The expression of MAPK1 was notably heightened in tumor tissues relative to matched normal tissues (Figure 5D), and MAPK1 mRNA expression was negatively correlated with miR‐149‐5p expression in tumor tissues (Figure 5E). The expression of MAPK1 protein was also markedly increased in tumor tissues (Figure 5F). As expected, MAPK1 protein level was significantly enhanced in SW579 and TPC‐1 cells compared with Nthy‐ori 3–1 cells (Figure 5G). Interestingly, MAPK1 protein expression was markedly declined in SW579 and TPC‐1 cells with miR‐149‐5p depletion but strengthened in SW579 and TPC‐1 cells with miR‐149‐5p overexpression (Figure 5H). The data revealed that MAPK1 was a target of miR‐149‐5p.

### 3.6 MiR‐149‐5p restoration‐inhibited thyroid cancer cell malignant development by sequestering MAPK1

**FIGURE 6:**
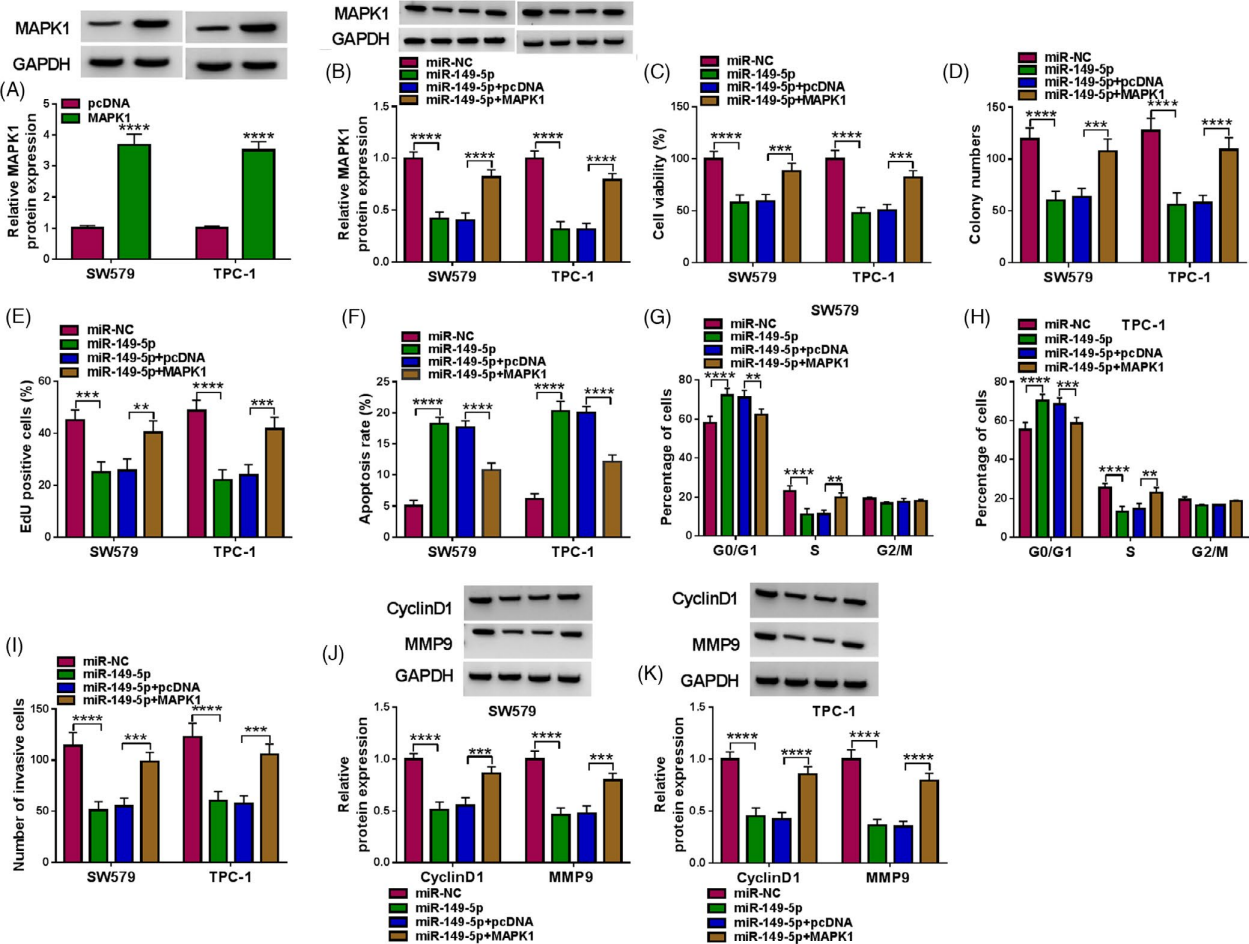
MiR‐149‐5p restoration‐suppressed thyroid cancer cell proliferation, survival, and invasion were restored by MAPK1 overexpression. (A) The efficiency of MAPK1 overexpression was checked by Western blot. (B) The expression of MAPK1 protein in SW579 and TPC‐1 cells transfected with miR‐149‐5p or miR‐149‐5p+MAPK1 was measured by Western blot. In these transfected cells, (C) cell viability was detected by CCK‐8 assay. (D) The ability of colony formation was detected by colony formation assay. (E) Cell proliferation was assessed by EdU assay. (F) Cell apoptosis was monitored by flow cytometry assay. (G and H) Cell cycle was determined by flow cytometry assay. (I) Cell invasion was determined by transwell assay. (J and K) The protein levels of CyclinD1 and MMP9 were measured by Western blot. **p < 0.01, ***p < 0.001 and ****p < 0.0001

To validate whether miR‐149‐5p played functions by targeting MAPK1, rescue experiments were performed. MAPK1 protein level was strikingly strengthened in SW579 and TPC‐1 cells transfected with MAPK1 compared with pcDNA (Figure 6A). For rescue experiments, the expression of MAPK1 was notably decreased in SW579 and TPC‐1 cells transfected with miR‐149‐5p but recovered in SW579 and TPC‐1 cells transfected with miR‐149‐5p+MAPK1 (Figure 6B). In function, cell viability was markedly suppressed in SW579 and TPC‐1 cells with miR‐149‐5p transfection but recovered in cells with miR‐149‐5p+MAPK1 transfection (Figure 6C). The ability of cell proliferation was notably impaired by miR‐149‐5p restoration but restored by the reintroduction of MAPK1 in SW549 and TPC‐1 cells (Figure 6D and 6E). In addition, miR‐149‐5p restoration‐induced SW549 and TPC‐1 cell apoptosis and cell cycle arrest (at G0/G1 stage) were substantially relieved by the reintroduction of MAPK1 (Figure 6F–6H). Moreover, the capacity of cell invasion in SW579 and TPC‐1 cells was suppressed by miR‐149‐5p enrichment but partially promoted by MAPK1 overexpression (Figure 6I). The expression levels of CyclinD1 and MMP9 were significantly lessened in SW579 and TPC‐1 cells with miR‐149‐5p transfection but largely enhanced in cells with miR‐149‐5p+MAPK1 cotransfection (Figure 6J and 6K). These data indicated that miR‐149‐5p restoration inhibited thyroid cancer cell malignant development by sequestering MAPK1.

### 3.7 **Circ‐CSNK1G1** **knockdown downregulated MAPK1 by releasing miR‐149‐5p**

**FIGURE 7:**
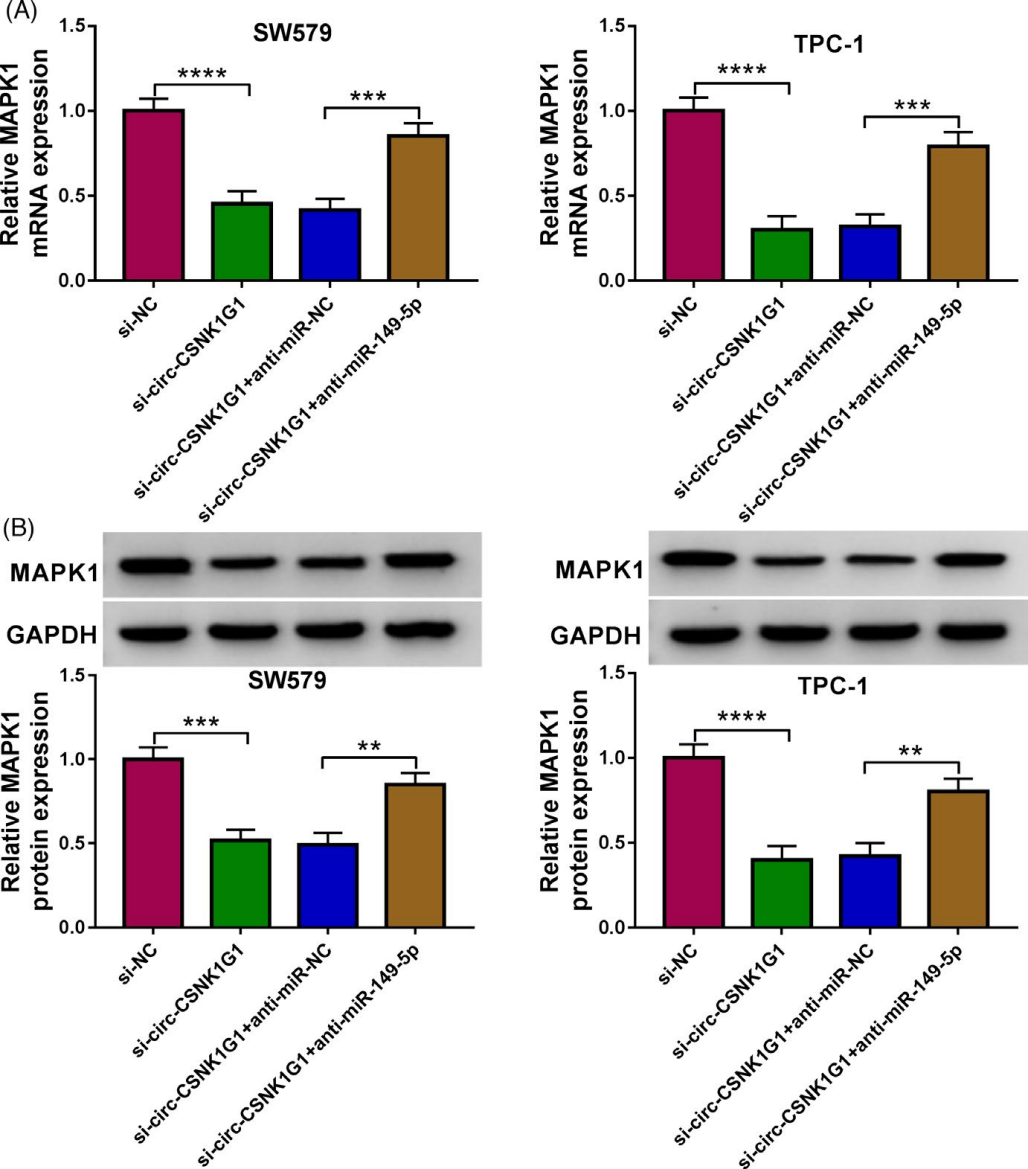
Circ‐CSNK1G1 knockdown inhibited the expression of MAPK1 by enriching miR‐149‐5p. (A and B) The expression of MAPK1 mRNA and protein in SW579 and TPC‐1 cells transfected with si‐circ‐CSNK1G1 or si‐circ‐CSNK1G1+anti‐miR‐149‐5p was detected by qPCR and Western blot. **p < 0.01, ***p < 0.001 and ****p < 0.0001

The expression level of MAPK1 mRNA and protein was significantly decreased in SW579 and TPC‐1 cells transfected with si‐circ‐CSNK1G1, while the level of MAPK1 mRNA and protein was remarkably increased in SW579 and TPC‐1 cells with si‐circ‐CSNK1G1+anti‐miR‐149‐5p cotransfection (Figure 7A and 7B), uncovering that circ‐CSNK1G1 governed the miR‐149‐5p/MAPK1 network.

### 3.8 The downregulation of circ‐CSNK1G1 inhibited tumor growth in animal models

**FIGURE 8:**
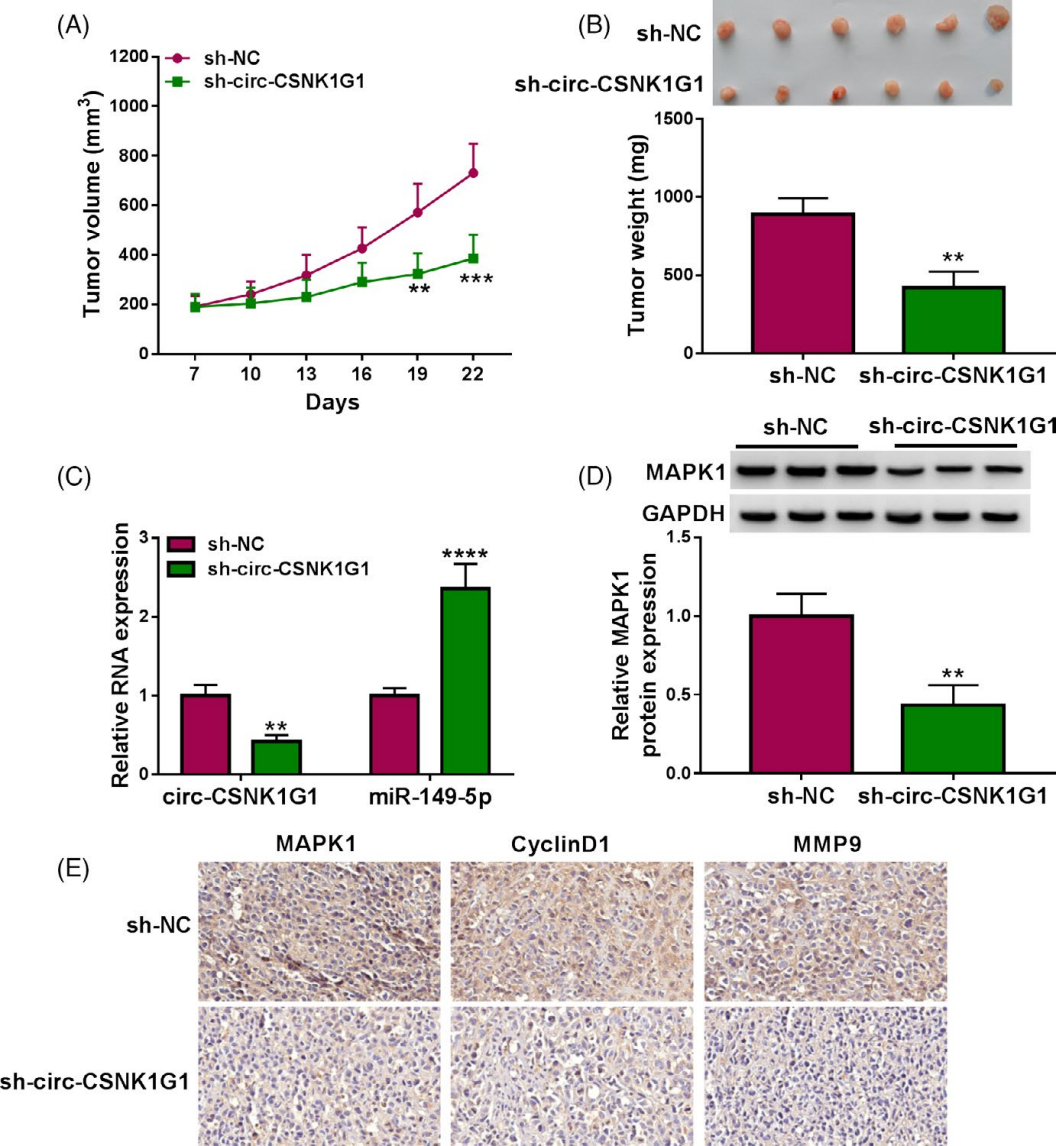
Circ‐CSNK1G1 knockdown inhibited tumor growth in animal models. (A and B) Tumor volume and tumor weight were measured to assess tumor growth. (C) The expression of circ‐CSNK1G1 and miR‐149‐5p in the excised tumor tissues from animal models was measured using qPCR. (D) The expression of MAPK1 protein in the excised tumor tissues from animal models was measured using Western blot. (E) The abundance of MAPK1, CyclinD1, and MMP9 in tumor tissues was investigated by IHC assay. **p < 0.01, ***p < 0.001 and ****p < 0.0001

We further confirmed the role of circ‐CSNK1G1 *in vivo*. Animal models were established in nude mice by subcutaneously injecting with TPC‐1 cells infected with sh‐circ‐CSNK1G1, using sh‐NC as a control. We found that tumor volume in the sh‐circ‐CSNK1G1 group was significantly lower than that in the sh‐NC group at the day 19 post‐injection (Figure 8A). Besides, circ‐CSNK1G1 knockdown inhibited tumor weight, leading to smaller tumor size (Figure 8B). Moreover, the data from qPCR showed that the expression of circ‐CSNK1G1 was decreased, while the expression of miR‐149‐5p was enhanced in sh‐circ‐CSNK1G1‐administered tumor tissues (Figure 8C). The expression of MAPK1 protein was pronouncedly declined in sh‐circ‐CSNK1G1‐administered tumor tissues (Figure 8D). Moreover, IHC analysis presented that the abundance of MAPK1, CyclinD1, and MMP9 was markedly decreased in tumor tissues from the sh‐circ‐CSNK1G1 group (Figure 8E). All data suggested that circ‐CSNK1G1 knockdown inhibited tumor growth and development through miR‐149‐5p‐mediated MAPK1 inhibition.

## 4 DISCUSSION

Our current data mainly found that circ‐CSNK1G1 was overexpressed in tumor tissues of thyroid cancer relative to normal tissues. The downregulation of circ‐CSNK1G1 inhibited cancer cell colony formation, proliferation, survival, and invasion, which supported that circ‐CSNK1G1 was a carcinogenic driver at least in thyroid cancer. Moreover, mechanism analysis suggested that circ‐CSNK1G1 played carcinogenic effects in this cancer by acting as miR‐149‐5p sponge and relieving the inhibition of miR‐149‐5p on MAPK1. These present findings deepened the insights into the realizing of the role of circ‐CSNK1G1 in thyroid cancer.

By reviewing the previous studies, we discovered that circ‐CSNK1G1 served as an oncogenic role to facilitate the malignant progression of diverse cancers.^14^, ^15^, ^16^ For example, circ‐CSNK1G1 level was increased in hepatocellular carcinoma (HCC) tumor tissues, and circ‐CSNK1G1 knockdown repressed tumor growth in animal model.^14^ In vitro assays showed that circ‐CSNK1G1 efficiently promoted HCC cell proliferation, invasion, and migration.^16^ The expression of circ‐CSNK1G1 was also aberrantly strengthened in colorectal cancer tissues.^15^ The evidence supported that cancer aggressive development might be associated with increased circ‐CSNK1G1 expression, whereas the role of circ‐CSNK1G1 in thyroid cancer was poorly investigated. Only a circRNA microarray profile presented that circ‐CSNK1G1 was one of the circRNAs that were significantly upregulated in papillary thyroid cancer tissues.^17^ We thus explored the detailed role of circ‐CSNK1G1 in thyroid cancer and found that circ‐CSNK1G1 downregulation suppressed the proliferation, survival, and invasion of cancer cells and tumor growth in vivo in thyroid cancer, which was consistent with the role of circ‐CSNK1G1 in other cancers. Moreover, the published studies have demonstrated the sponge effects of circ‐CSNK1G1 on certain miRNAs, such as miR‐516a‐5p and miR‐145‐5p.^14^, ^16^ Following this idea, we investigated the potential miRNAs targeted by circ‐CSNK1G1 to explore the functional mechanism of circ‐CSNK1G1.

Here, miR‐149‐5p was ensured as a target of circ‐CSNK1G1. MiR‐149‐5p has been widely investigated in different cancers, such as renal cell carcinoma, osteosarcoma, and HCC.^18^, ^19^, ^20^ In these cancers, miR‐149‐5p exerted anti‐tumor roles to block cancer cell growth and migration.^18^, ^19^, ^20^ Noticeably, miR‐149‐5p expression was lessened in papillary and medullary thyroid cancer tissues.^11,21^ The restoration of miR‐149‐5p was shown to obstruct the proliferation and invasion of medullary thyroid cancer cells by suppressing its target genes.^11^ In agreement with these findings, we summarized that miR‐149‐5p repression recovered circ‐CSNK1G1 knockdown‐blocked cell proliferation, survival, and invasion, while miR‐149‐5p restoration suppressed these cell malignant behaviors, exposing that miR‐149‐5p played a tumor‐suppressor role in thyroid cancer.

It is well known that miRNAs mediate translational inhibition of target genes in post‐transcriptional gene expression.^22^ Our study discovered that miR‐149‐5p targeted MAPK1 3'UTR, suggesting that MAPK1 was a target of miR‐149‐5p. Previous studies exhibited that MAPK1 overexpression promoted cell proliferation, glycolysis, and motility in papillary thyroid cancer.^12^, ^23^, ^24^ Our study found that the inhibitory proliferation, survival, and invasion of cancer cells caused by miR‐149‐5p restoration were largely restored by MAPK1 overexpression, indicating that miR‐149‐5p inhibited thyroid cancer progression by depleting MAPK1. The carcinogenic effects of MAPK1 were widely proposed in diverse cancers, such as gastric cancer and breast cancer.^25^, ^26^ These studies revealed that MAPK1 was an oncogene in cancers, and miR‐149‐5p bound to MAPK1 3'UTR, and thus inhibited MAPK1 expression. However, circ‐CSNK1G1 acted as a ceRNA to compete for miR‐149‐5p with MAPK1, thereby relieving the suppression of miR‐149‐5p on MAPK1.

Taken together, this study was the first study to exploit the detailed functions of circ‐CSNK1G1 in thyroid cancer. Circ‐CSNK1G1 downregulation restrained the proliferation, colony formation, survival, and invasion of thyroid cancer cells in vitro and tumor development in vivo. Circ‐CSNK1G1 promoted the malignant development of thyroid cancer partially by activating MAPK1 via competitively targeting miR‐149‐5p. Our study provides a basis for characterizing the role of circ‐CSNK1G1 in thyroid cancer.

## CONFLICT OF INTEREST

The authors declare that they have no conflict of interest.

## Data Availability

The datasets used and analyzed during the current study are available from the corresponding author on reasonable request.
